# Rare Angina: A Case Report of Ludwig's Angina

**DOI:** 10.7759/cureus.25873

**Published:** 2022-06-12

**Authors:** Anuradha Sakhuja, Dhan B Shrestha, Barun B Aryal, Wasey Ali Yadullahi Mir, Larissa Verda

**Affiliations:** 1 Department of Internal Medicine, Mount Sinai Hospital, Chicago, USA; 2 Department of Emergency Medicine, B.P. Smriti Hospital, Kathmandu, NPL

**Keywords:** ludwig's angina, debridement, anti-bacterial agents, cellulitis, neck

## Abstract

Ludwig’s angina is the rapidly progressive cellulitis of the soft tissue of the neck and the floor of the mouth. Airway compromise is a frequent and potentially fatal sequela of Ludwig’s angina. Here we present a case of a 54-year-old African American male who presented with fever associated with painful swelling of the mouth and anterior neck. He was febrile and hypoxic on presentation. Imaging showed extensive involvement of the neck and mediastinum to the level of the clavicles. The diagnosis of Ludwig’s angina of periodontal origin was made, and intubation was performed for airway protection. Management was done by surgical debridement along with a course of broad-spectrum antibiotics. The patient’s condition improved, and he was discharged on oral antibiotics with a referral to a dentist. Our case demonstrates that early diagnosis, airway management, treatment with broad-spectrum antibiotics, and surgical intervention are vital for the successful management of severe cases of Ludwig’s angina.

## Introduction

Ludwig’s angina is a rapidly spreading and often fatal progressive cellulitis of soft tissues of the neck and floor of the mouth. It was first described in 1836, and before the widespread use of antibiotics, more than 50% of the cases were fatal [1,2]. The infection is odontogenic in 85% of cases, and other causes include peritonsillar abscess, parapharyngeal abscess, mandibular fractures, oral piercings or wounds, and submandibular sialadenitis [3,4]. Most documented cases occur in adult males 20 to 60 years of age [4-11]. The infection can spread contiguously to the sublingual, pharyngomaxillary, and retropharyngeal spaces. Rapidly progressive swelling of the soft tissue of the neck and posterior displacement of the tongue can occur, predisposing to life-threatening airway obstruction. Diagnosis is usually clinical, and treatment includes high-dose antibiotics, steroids, surgical debridement, and supportive measures. Here, we present the case of a previously healthy 54-year-old male who was diagnosed with Ludwig’s angina.

## Case presentation

A 54-year-old African American male with a past medical history of hypertension, heart failure with reduced ejection fraction (20%-25%), chronic obstructive pulmonary disease (COPD) on inhalers presented with a two-week history of right mouth/jaw pain, foul taste, and one week of anterior neck swelling. The jaw pain was also associated with difficulty in opening the mouth. He was febrile at 102^o^f, tachycardic to 140, normotensive, and oxygen saturation of 92% on room air at presentation. Physical examination was significant for very poor dentition and swelling of the lower jaw. The jaw swelling was extended to his neck, overlying the clavicles laterally, and associated with diffuse tenderness to palpation over the sublingual, submandibular, and anterior neck regions. Prominent trismus and crepitus in the anterior upper chest wall were also noted. Initially, the patient was stabilized and managed symptomatically with analgesics, antipyretics, and oxygen supplementation. His laboratory indicated marked leukocytosis with a neutrophilic predominance (71%) and bandemia (13%). 

**Figure 1:**
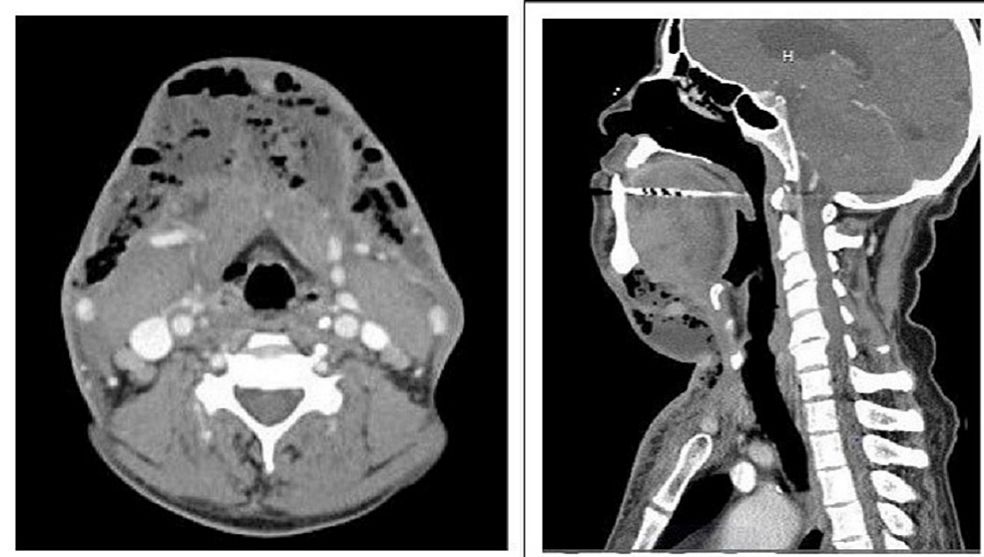
CT scan of neck showing left sublingual space 2.5x0.8 cm fluid collection, submandibular space collection 2x2.1 cm

Human immunodeficiency virus (HIV) and Hepatitis C virus (HCV) were negative. Computed tomography (CT) scan of the neck with contrast showed left sublingual space (2.5x0.8 cm) fluid collection, submandibular space collection of 2x2.1 cm (Figure 1) extending to subcutaneous fat of the neck (4x8.7 cm), extending caudally to the anterior chest wall, infiltrating mediastinum at the level of clavicles, all originating from right mandibular premolar, concerning for Ludwigs's angina. It also showed extensive dental caries throughout mandibular premolars and subcutaneous emphysema of the right supraclavicular region. He was immediately started on broad-spectrum antibiotics after blood cultures were drawn. Given the high risk of airway compromise from extensive inflammation and potential airway obstruction, he was intubated and placed on positive pressure ventilation. He was immediately taken to surgery for the debridement and drainage of the anterior neck, submental area, the floor of the mouth, bilateral submandibular space, and the anterior chest wall. Multiple drains were placed at that time. Overall, the patient did clinically improve on antibiotics but had persistent drainage from the chest drains and started to develop superficial erythema of the infraclavicular anterior chest wall area. Hence, the CT scan of the neck and chest was repeated, which showed a clear mediastinum; however, it had abscess pockets along the anterior chest wall that required further debridement and drainage. Intraoperatively, it appeared to have loculations upon palpation along the anterior chest wall both laterally and medially. These loculations were broken up digitally, and volumes of pus were noted along. Later during the hospital course, he showed a decrease in neck swelling and clinically improved. He was successfully extubated and transitioned to oral antibiotics, discharged with the continued antibiotic course for three weeks, and referred to oral surgery services for tooth extraction and oral care.

## Discussion

Wilhelm Frederick von Ludwig first described Ludwig’s angina in 1836 as progressive swelling of the soft tissues of the floor of the mouth and neck [1]. The submandibular spaces are the most commonly affected, but the infection can spread to surrounding neck spaces. Airway obstruction is the most feared complication of Ludwig’s angina and the most common cause of mortality. This can be due to the displacement of the floor of the mouth and the tongue posteriorly or the involvement of deeper neck spaces like the retropharyngeal, pretracheal, and lateral pharyngeal spaces. The involvement of mediastinum and pericardium is less common but reported [5,7]. Infection is often polymicrobial, with Streptococcus pyogenes, Peptostreptococci, and Bacteroides being the most commonly implicated organisms [11].

A six-year retrospective study of 6092 ICU patients in China found that only 33 patients were admitted with Ludwig’s angina [12]. Out of them, 29 were included in the study, among which 97% required mechanical ventilation, all of them required surgical debridement, and 10% of them died. This shows that while Ludwig’s angina is rare, it is life-threatening and needs intensive management. Our case is a typical presentation of Ludwig’s angina: a male between 20 and 60 years presenting with fever, mouth and anterior neck pain, and swelling [3]. The dental origin of Ludwig’s angina, as was seen in our case, is by far the most commonly reported cause [3,13]. However, the extensive involvement of the anterior neck extends up to the mediastinum, which is relatively uncommon.

Moreover, our patient was hypoxic on arrival at the hospital and required intubation, signifying a severe presentation. As there was marked edema and swelling, early intubation was done with the involvement of an anesthetist and an otolaryngologist for airway protection. Given the extensive involvement of the neck, urgent surgical debridement was performed to drain the abscess and reduce the swelling.

The management of Ludwig’s angina is based on the principles of airway protection and treatment of the infection. A retrospective study of emergency department visits in the United States between 2006 and 2014 showed that among 5855 patients with Ludwig’s angina, 47% required surgical debridement [14]. Our patient required repeated surgical debridement, antibiotics, and endotracheal intubation to prevent airway obstruction. Repeat debridement was needed because of the lack of complete resolution of swelling. In cases with a dental origin of the infection identified early on, extraction of the tooth/teeth involved is necessary to remove the source of infection [13]. However, in our case, extensive involvement of the neck spaces with fluid collection on imaging meant that surgical incision and drainage took precedence over tooth extraction due to the risk of life-threatening airway compromise. Therefore, the choice of airway management in Ludwig’s angina depends on individual patients' neck and airway findings. Generally, tracheostomy using local anesthesia is considered the gold standard in patients with deep neck infections. Still, the anatomical distortion caused by swelling can make it quite difficult in patients with severe infection and extensive involvement of the neck spaces. Our patient was successfully managed with intubation.

Although mortality of Ludwig’s angina has decreased significantly over the years, it still poses a challenge to clinicians because of its rarity and high likelihood of airway compromise. Nevertheless, our case demonstrates that with timely intervention to protect the airway and resolve the infection, even severe cases of Ludwig’s angina can have a good outcome.

## Conclusions

Although rare, Ludwig’s angina is a life-threatening condition. Poor oral hygiene can lead to Ludwig's Angina. Early diagnosis, airway management, treatment with broad-spectrum antibiotics, and surgical intervention are vital for successfully managing Ludwig’s angina with extensive soft tissue involvement.
